# Supplementary material for: Persister state-directed transitioning and vulnerability in melanoma
Source: Nat Commun. 2022 Jun 1;13:3055. doi: 10.1038/s41467-022-30641-9 (PMC9160289; doi:10.1038/s41467-022-30641-9)
Supplement: Supplementary file 1 — Supplementary Information [file 41467_2022_30641_MOESM1_ESM.pdf]

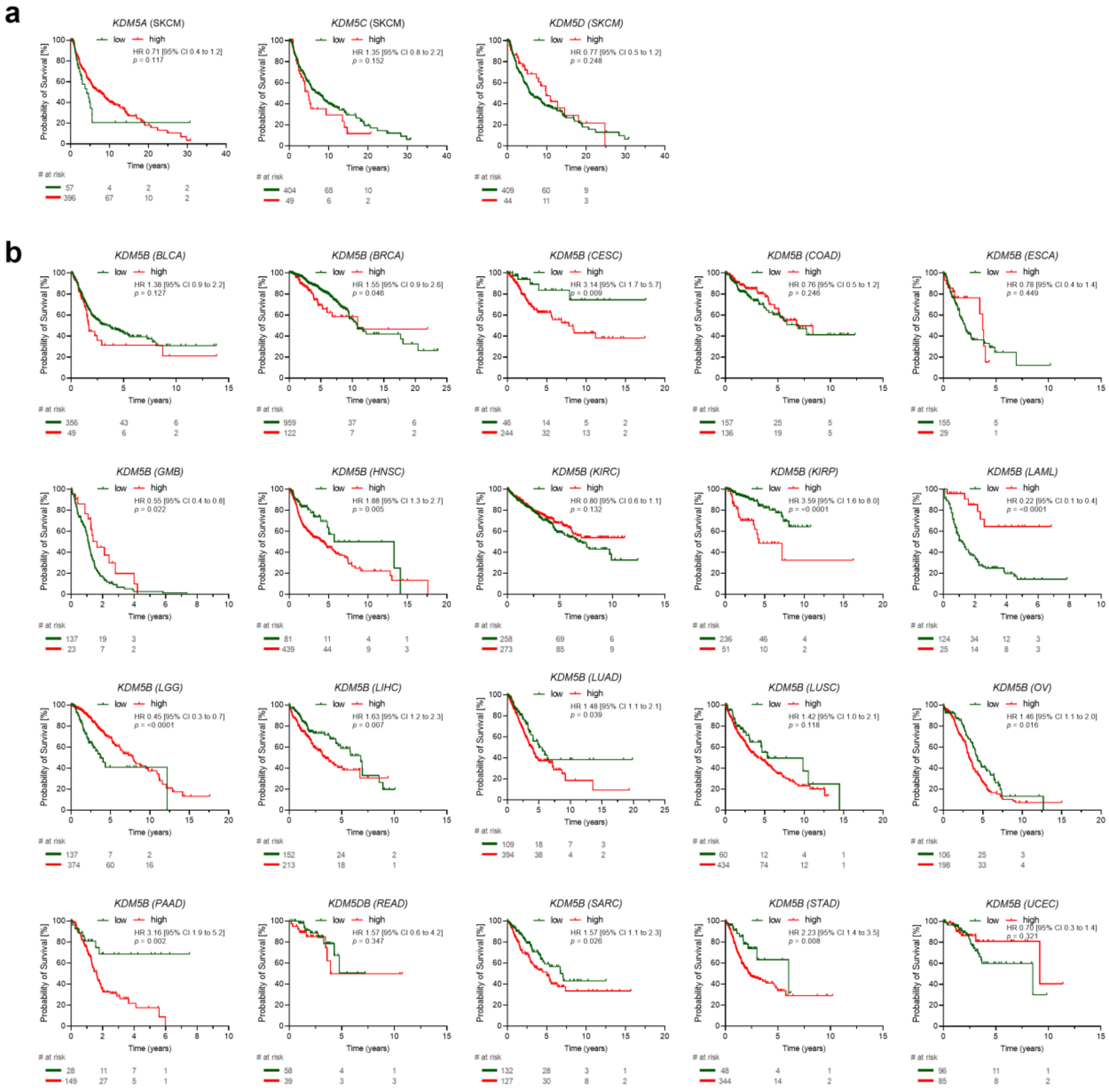

Supplementary Figure 1. KDM5 gene-dependent patient overall survival across different cancer entities, Related to Figure 1. (**a**) Kaplan-Meier survival curves of melanoma patients for KDM5A, KDM5C, and KDM5D. (**b**) Kaplan-Meier survival curves for KDM5B across different cancer entities. Curves were calculated from the TCGA data set based on cut-point optimization to distinguish expression into low and high expression (high expression, red, vs. low, green). Graphs were created by the UCSC Xena, X-Tile and GraphPad Prism. Sample sizes are indicated in the patient at risk table (# of risk). Significance was tested by Long-rank (Mantel-Cox) test. Source data are provided as a Source Data file.

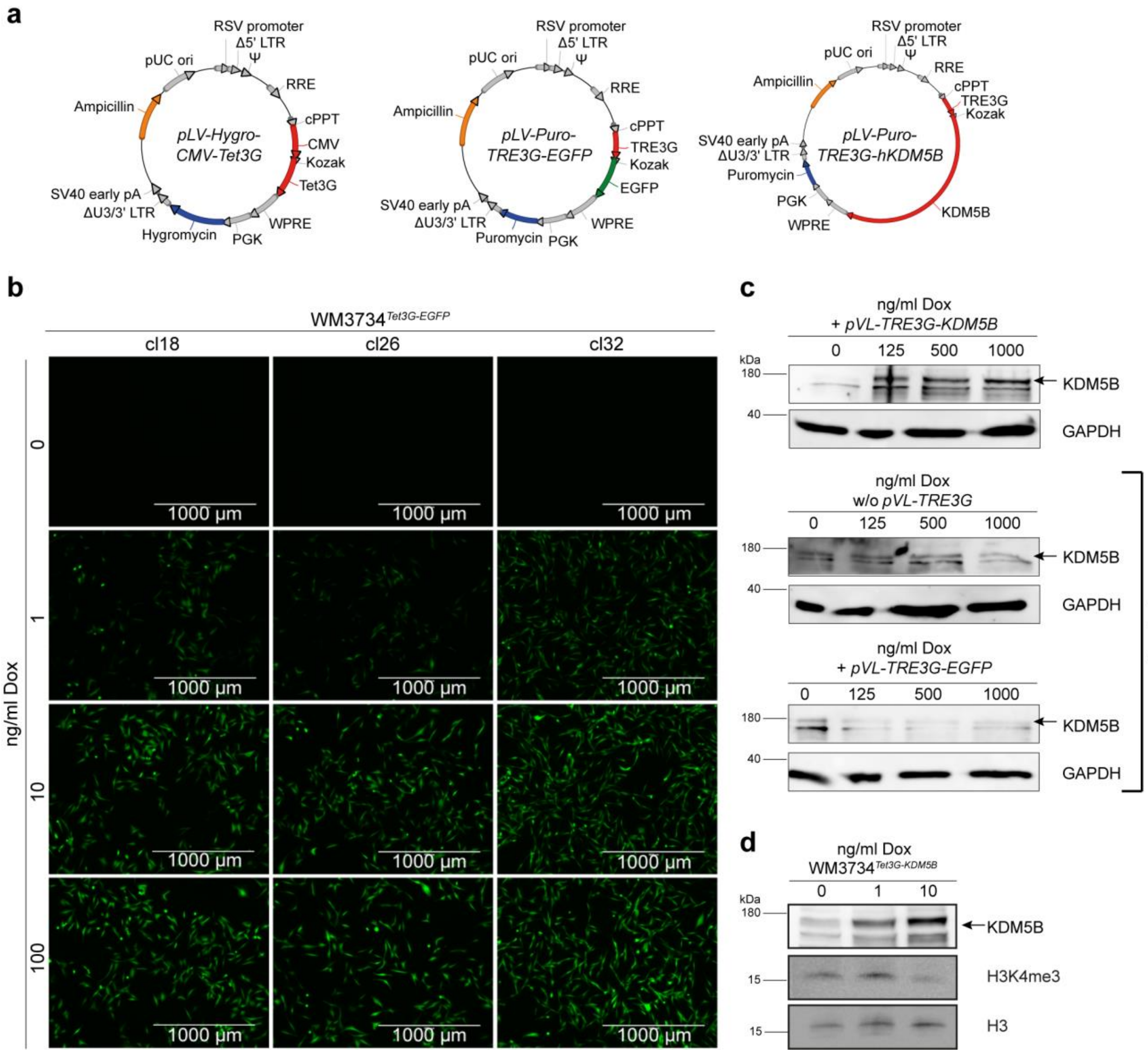

Supplementary Figure 2. Establishment of a doxycycline-inducible Tet-On 3G-system for KDM5B protein overexpression, Related to Figure 1. (**a**) Vector maps of the plasmid encoding the CMV promoter-driven Tet3G (left, pLV-Hygro-CMV-Tet3G), the TRE response vector with an inducible P_{TRE3G} promoter plus the control gene EGFP (middle, pLV-Puro-TRE3G-EGFP), and the TRE response vector with an inducible P_{TRE3G} promoter plus the human KDM5B gene (right, pLV-Puro-TRE3G-hKDM5B, transcript variant 1, NM_001314042.1). (**b**) EGFP protein induction at increasing Dox concentrations in three different clones of stably transduced WM3734$^{Tet3G\text{-}EGFP}$ control cells imaged after 48 h of induction (n=3). (**c**) Immunoblotting of KDM5B protein induction in WM3734^{Tet3G} cells transduced with pVL-TRE3G-KDM5B at different Dox levels (top panel). Dox was titrated up to 1000 ng/ml to exclude cell toxicity (n=1). Absence of KDM5B protein induction in WM3734^{Tet3G} control cells (middle) and WM3734$^{Tet3G\text{-}EGFP}$ control cells (bottom). (**d**) Representative immunoblots of KDM5B and histone H3K4me3 after 22 days of Dox induction in WM3734$^{Tet3G\text{-}KDM5B}$ cells (n=2). Source data are provided as a Source Data file.

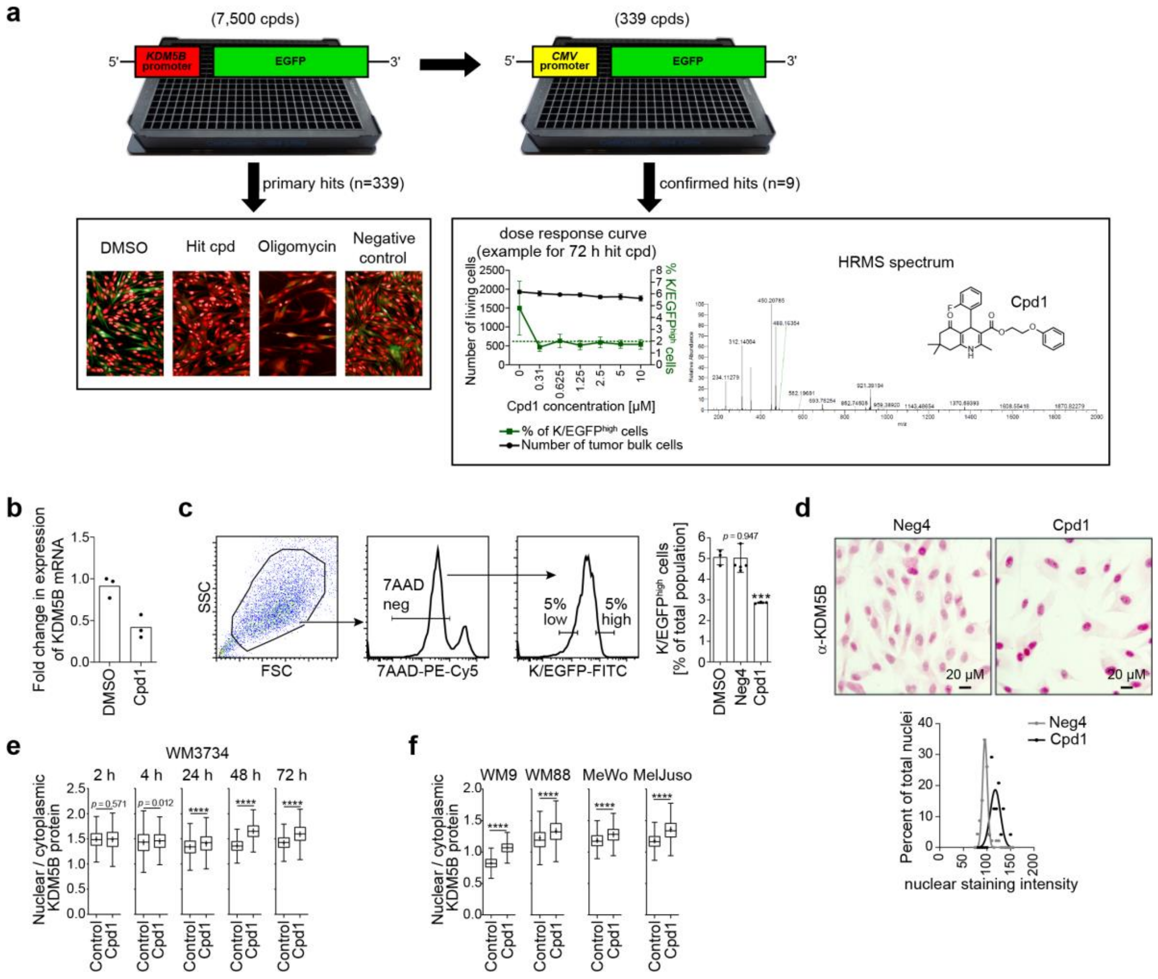

Supplementary Figure 3. Screening for small chemical compounds that modulate KDM5B expression, Related to Figure 1. (**a**) Schematic of the phenotypic compound screening assay consisting of a primary screen based on the WM3734$^{KDM5Bprom\text{-}EGFP}$ cell model and a counter screen with WM3734$^{CMVprom\text{-}EGPF}$ control cells performed on an Opera High Content Screening system. Dose response curves were done for confirmed hit compounds, as e.g. compound no. 1 (abbreviated Cpd1). The structural formula and HRMS purity analysis of Cpd1 is shown at the right. For more details, see methods. (**b**) Quantitative PCR detection of endogenous KDM5B mRNA transcripts (all isoforms covered) after 72 h of Cpd1 treatment in WM3734 cells. Shown is one representative experiment (mean, n=4). (**c**) Independent hit validation by flow cytometric detection showed reduced KDM5B-promoter-driven EGFP (K/EGFP) expression in WM3734 cells after 72 h of treatment with the hit compound Cpd1 (10 µM) compared to DMSO and the structural analog compound (Molecular ACCess System (MACCS) similarity of 0.899) Neg4 (10 µM), which lacks comparable activity. Mean ±SD (n=3); two-sided t-test (***p=0.0005). (**d**) Anti-KDM5B nuclear immunostaining of WM3734 melanoma cells after 72 h of Cpd1 vs. Neg4 treatment (10 µM). Left, representative pictures; right, quantitation shown as normalised frequency distribution of nuclear staining intensity. (**e**) Digital microscopic quantitation of nuclear vs. cytoplasmic KDM5B protein expression in WM3734 cells after Cpd1 treatment or DMSO as control over time and (**f**) across different melanoma cell lines (WM9, WM88, MeWo, MelJuso) after 72 h of treatment. Mean ±SD; ****p≤0.0001 by two-sided t-test. Box-and-whiskers represent median values and interquartile range; the mean values are plotted as crosses. Shown are results from 2-4 independent replicates with at least 3 quantified images per experiment. Source data are provided as a Source Data file.

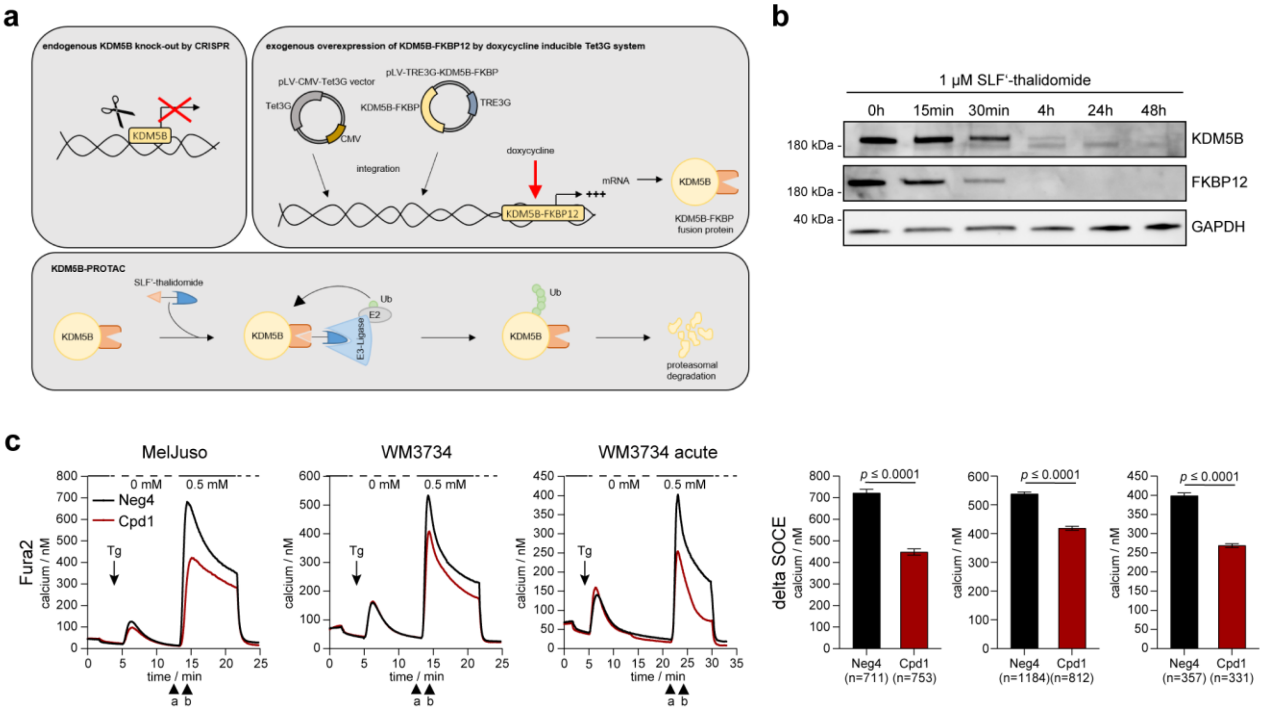

Supplementary Figure 4. Ca^{2+} dependent proteasomal degradation of KDM5B, Related to Figure 1. (**a**) Schematic of the KDM5B proteolysis targeting chimera (PROTAC) model. Heterobifunctional SLF'-thalidomide is used for directed E3 ligase-dependent protein degradation. The exogenous KDM5B-FKBP12 fusion protein is expressed from a stably integrated doxycycline inducible Tet3G expression plasmid system, while endogenous KDM5B is genetically knocked-out by CRISPR/Cas9. The SLF' domain of the PROTAC molecule binds to FKBP, while the thalidomide domain recruits the E3 ubiquitin ligase cereblon (CRBN) for ubiquitination and subsequent proteosomal degradation of the target protein KDM5B. (**b**) Time course of KDM5B protein levels upon induction of proteasomal degradation with SLF'-thalidomide in the KDM5B-PROTAC model. Shown is one representative experiment (n=3). (**c**) Fura-2-based Ca^{2+} imaging of MelJuso and WM3734 cells after 72 h of 10 µM Cpd1 or as control Neg4 treatment or with acute addition of the treatment during the experiment ('acute') was measured in Ringer's buffer containing 0.5 mM Ca^{2+} (solid line). ER-store depletion was initiated by 1 µM thapsigargin (Tg) in the absence of Ca^{2+} (dotted line); Store-operated calcium entry (SOCE) was measured upon re-addition of 0.5 mM Ca^{2+}. Left panels show the measured calcium concentrations over time, the right panels show the quantification of delta SOCE (b-a, as indicated in the figure presented as mean ± SEM (n-values: MelJuso: Neg4=711, Cpd1=753; WM3734: Neg4=1184, Cpd1=812; WM3734 'acute' Neg4=357, Cpd1=331). Statistical significance was addressed using unpaired, two-sided Student's t-test. Source data are provided as a Source Data file.

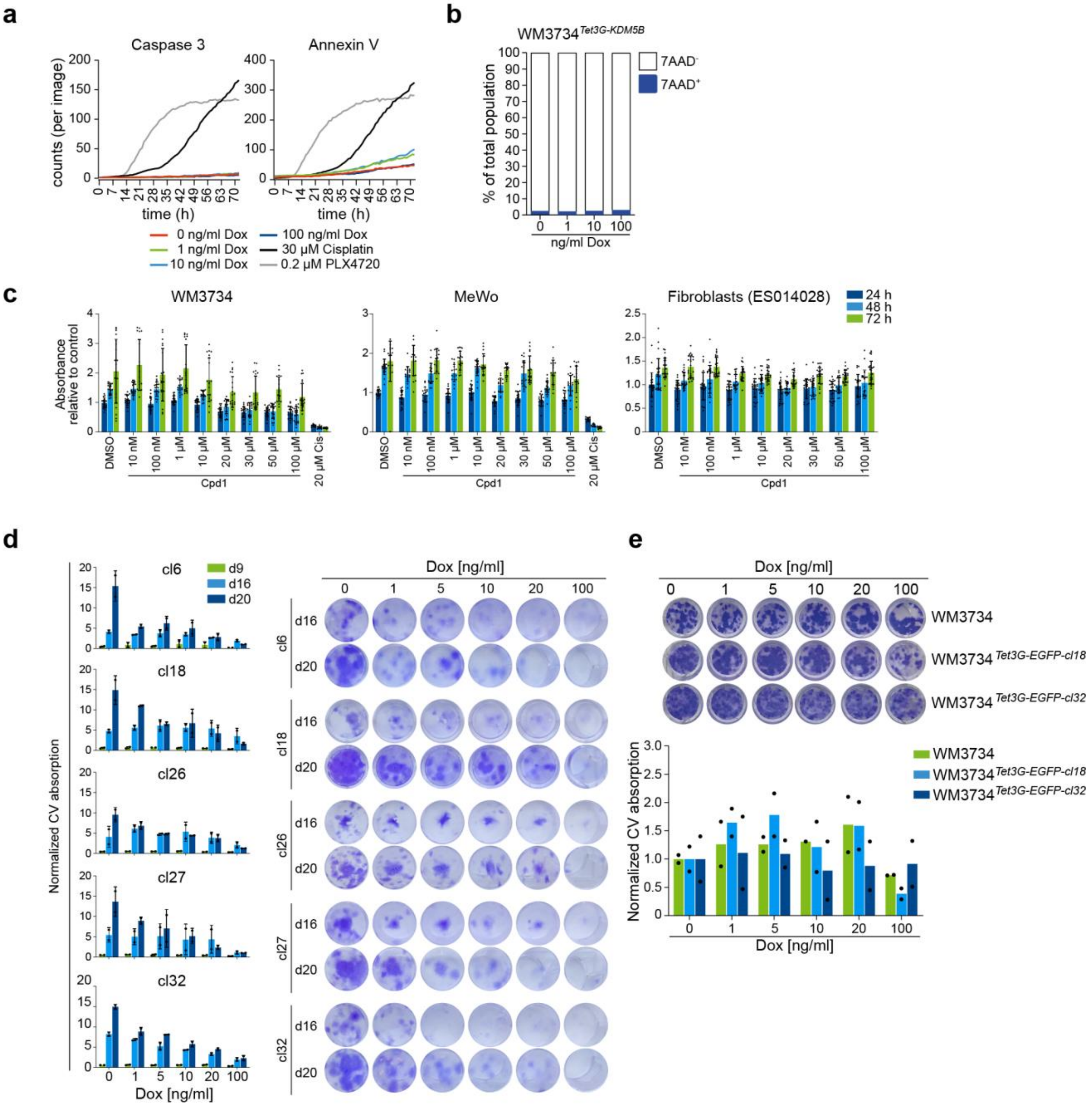

Supplementary Figure 5. *In vitro* effects of enforced KDM5B expression, Related to Figure 2. (**a**) Caspase 3 and annexin V detection over 72 h determined by IncuCyte analysis. Cisplatin and PLX4720 were used as positive controls for induction of apoptosis. Shown is one representative experiment (n=3). (**b**) Flow cytometric determination of 7AAD^{+} dead cells after 48 h of KDM5B induction by Dox. Shown is one representative experiment (n=4). (**c**) MTT assay after 24, 48, and 72 h of Cpd1 treatment in WM3734 and MeWo melanoma cells compared to primary ES014028 fibroblasts. Mean ±SD (n=3). (**d**) Long-term clonogenic growth assay after gradual KDM5B induction over 9, 16, and 20 days with Dox from 5 different WM3734$^{Tet3G\text{-}KDM5B}$ clones (left, crystal violet quantitation; right, representative pictures from day 16 and 20). Mean (n=1). (**e**) Clonogenic long-term growth of WM3734$^{Tet3G\text{-}EGFP}$ control clones and naïve WM3734 cells after 20 days of Dox exposure (upper panel, representative pictures; lower panel, crystal violet quantitation). Shown is one representative experiment out of 2 independent replicates. Source data are provided as a Source Data file.

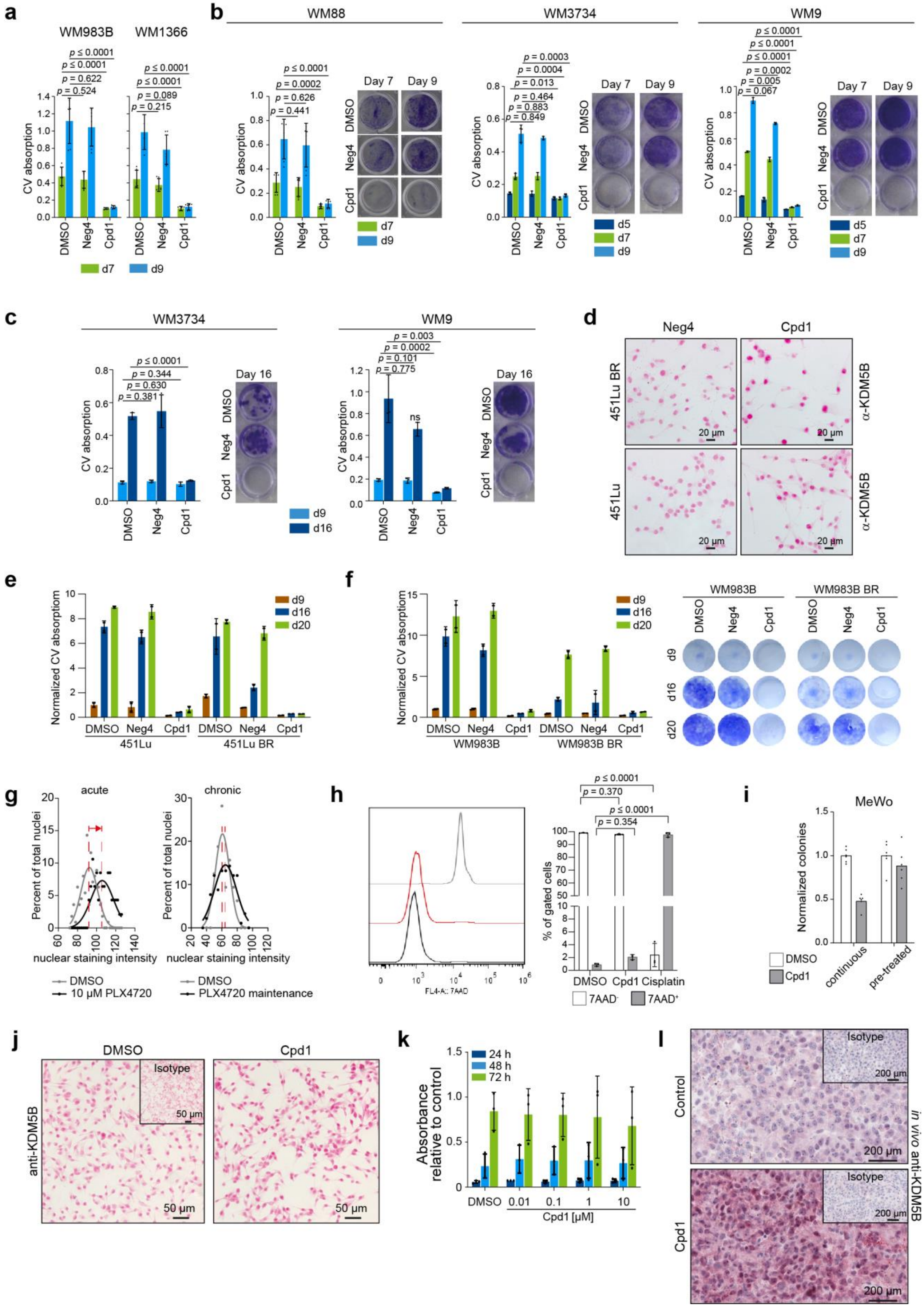
a
WM983B
WM1366
CV absorption
DMSO
Neg4
Cpd1
d7
d9
b
WM88
WM3734
WM9
Day 7
Day 9
d5
c
Day 16
d16
d
Neg4
Cpd1
451Lu BR
451Lu
α-KDM5B
20 µm
e
Normalized CV absorption
d20
f
WM983B BR
g
acute
chronic
Percent of total nuclei
nuclear staining intensity
10 µM PLX4720
PLX4720 maintenance
h
% of gated cells
Cisplatin
i
MeWo
Normalized colonies
continuous
pre-treated
j
anti-KDM5B
Isotype
50 µm
k
Absorbance relative to control
24 h
48 h
72 h
Cpd1 [µM]
l
Control
in vivo anti-KDM5B
200 µm

Supplementary Figure 6. *In vitro* and *in vivo* effects of enforced KDM5B expression, Related to Figure 2. (**a**) Quantitation of clonogenic growth assay of Fig. 2E. Shown is one representative experiment (mean ±SD, n=3; two-sided t-test). (**b**) Clonogenic growth assay of WM88, WM3734 and WM9 cells continuously treated over 5, 7 and 9 days with 10 µM of Cpd1 or Neg4 (left, crystal violet quantitation; right representative pictures from day 7 and 9). Shown is one representative experiment (mean ±SD, n=3; two-sided t-test). (**c**) Clonogenic growth assay of WM3734 and WM9 cells continuously treated over 9-16 days with 10 µM of Cpd1 or Neg4 (left, crystal violet quantitation (mean ±SD, n=1 for WM3734 and n=3 for WM9; two-sided t-test); right representative images from day 16). (**d**) Representative images of anti-KDM5B immunostaining of chronically PLX4720-resistant 451Lu BR vs. treatment-naïve 451Lu melanoma cells (n=2). Cells were treated for 72 h with Cpd1 (10 µM) or Neg4 (10 µM). (**e**) Quantitation of clonogenic growth assay of 451Lu and 451Lu BR cells shown in Fig. 2F (mean ±SD, n=2). (**f**) Clonogenic growth assay of WM983B and PLX4720-resistant WM983B-BR cells treated over 9,16 and 20 days with 10 µM of Cpd1 or Neg4 (left, crystal violet quantitation, mean ±SD, n=1; right, representative pictures). (**g**) Quantitated anti-KDM5B immunostaining shown as normalised frequency distribution of nuclear staining intensity. Treatment-naïve 451Lu melanoma cells were exposed to 10 µM PLX4720 over 72 h and compared to the DMSO control (left panel). Chronically resistant 451LuBR cells were maintained under the presence of 1 µM PLX4720 and compared to naïve 451Lu under DMSO treatment (right panel). (**h**) Left panel, representative histograms of 7AAD staining after 72h of DMSO (black), 10 µM Cpd1 (red) or as control 20 µM cisplatin (grey) treatment. Right panel, quantitation of 7AAD signals after 72 h of treatment (mean ±SD, n=3; two-way ANOVA-test). (**i**) Soft agar colony formation of MeWo cells under constant Cpd1 treatment or after pre-treatment with Cpd1 for 72 h before seeding. Shown are results of 2 independent experiments with each being performed in triplicate reaction. (**j**) Anti-KDM5B nuclear immunostaining of CM melanoma cells after 72 h of *in vitro* Cpd1 or as control DMSO treatment. (**k**) MTT assay after 24, 48, and 72 h of Cpd1 treatment in CM cells (mean ±SD, n=3). (**l**) Anti-KDM5B nuclear immunostaining of CM melanoma tumor grafts from Cpd1-treated mice and control mice. Representative images of n=3 per control and n=5 mice per treatment group. Source data are provided as a Source Data file.

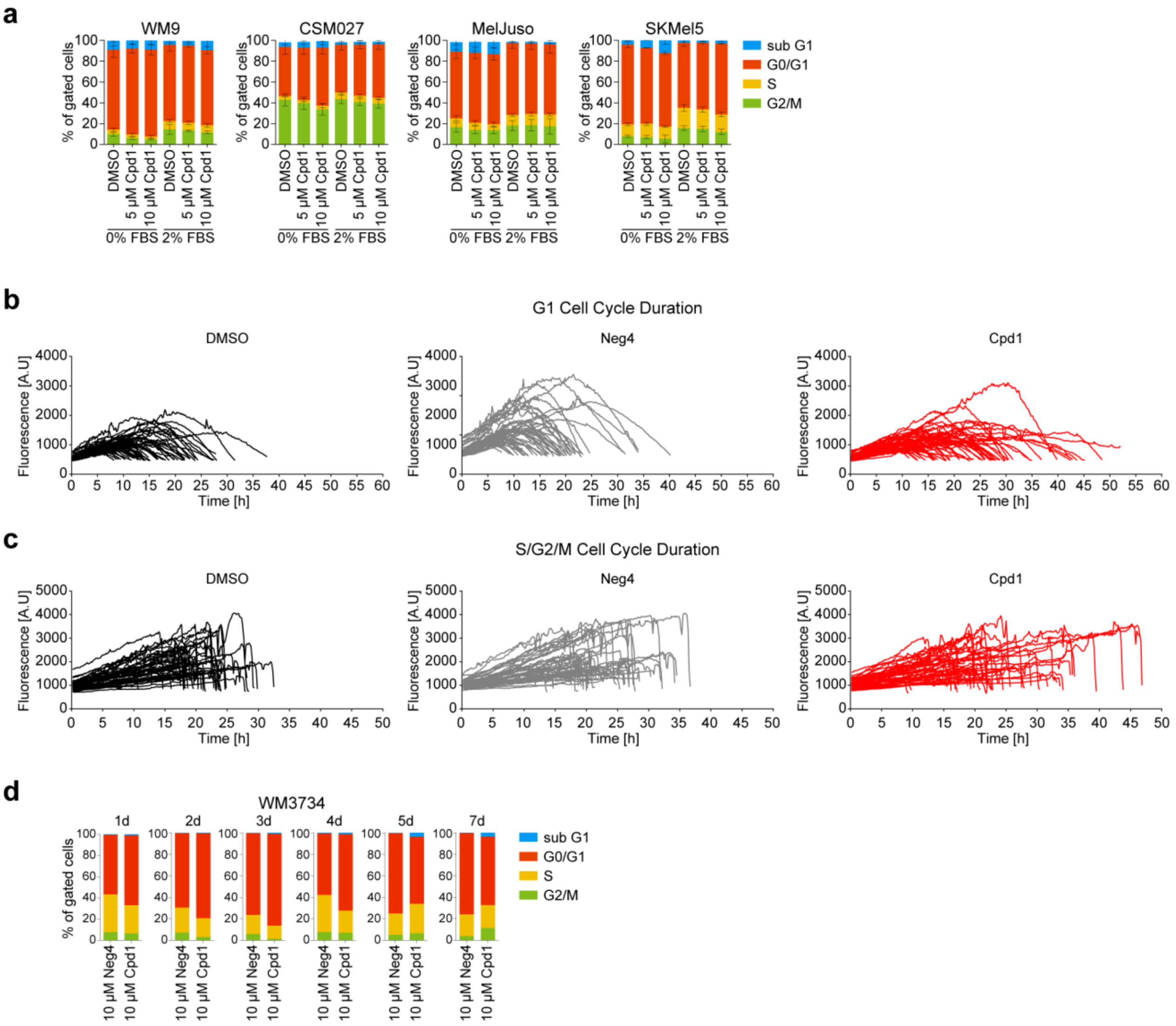

Supplementary Figure 7. Effects of enforced KDM5B expression on cell cycle, Related to Figure 3. (**a**) Propidium iodide flow cytometric cell cycle analysis after 72 h of Cpd1. WM9, CSM027, MelJuso, and SKMel5 cells were either continuously starved (0% FBS) or starved and then released by 2% FBS. Mean ±SD (n=4). (**b** and **c**) G1 (**b**) and S/G2/M (**c**) cell cycle duration of single cells (n=49) measured by FUCCI time-lapse imaging after 72 h of treatment with Cpd1 (10 µM) vs. DMSO or Neg4 (10 µM) controls. (**d**) Propidium iodide flow cytometric cell cycle analysis up to 7 days of Cpd1 treatment in WM3734 cells (n=3). Source data are provided as a Source Data file.

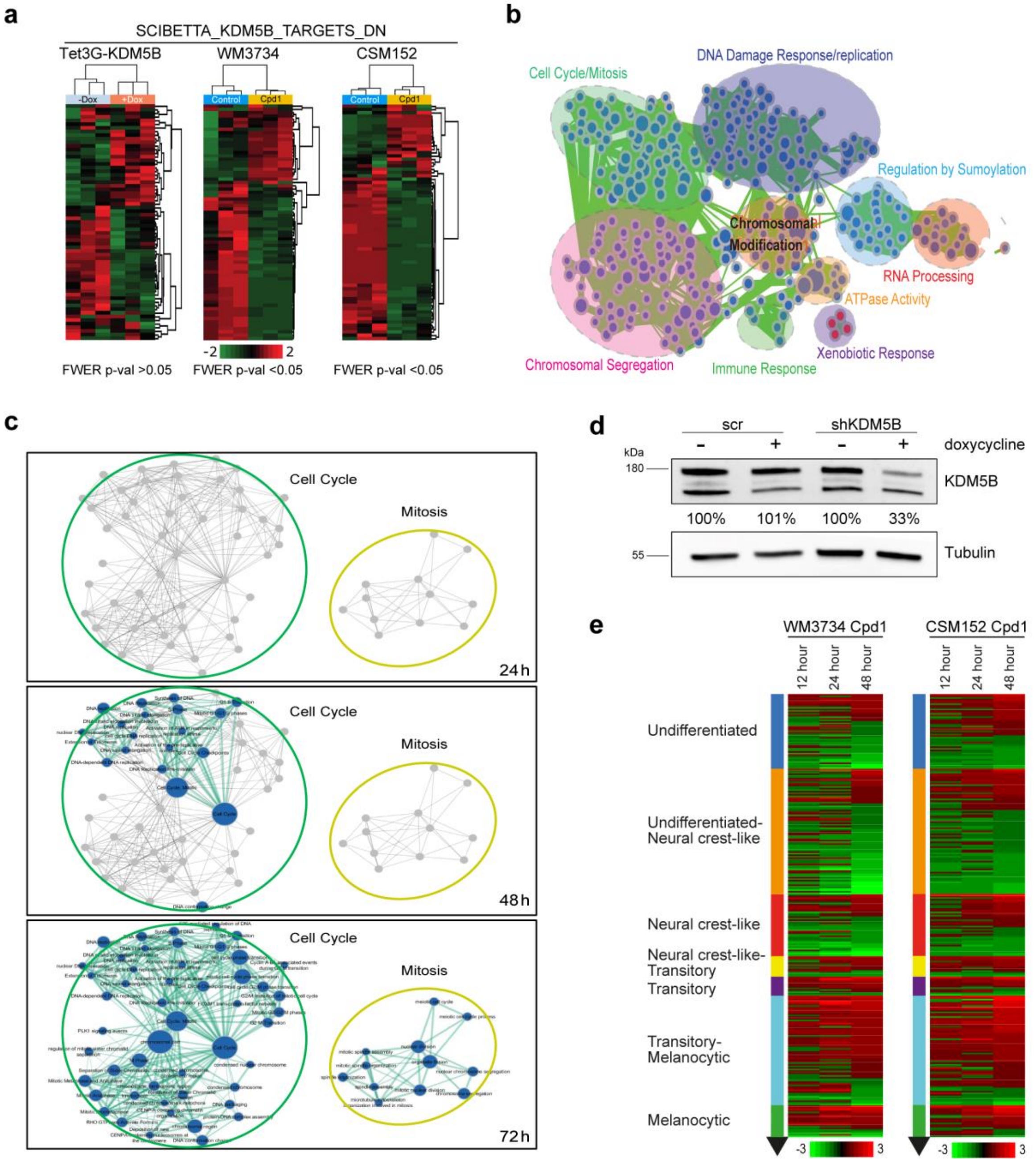

Supplementary Figure 8. Transcriptional shifts as a result of enforced KDM5B expression, Related to Figure 4. (**a**) Heatmaps of the SCIBETTA_KDM5B_TARGETS_DN (DN='down') motif [1] from WM3734$^{Tet3G\text{-}KDM5B}$ cells after 72 h of Dox treatment compared to corresponding WM3734 cells and short-term cultured CSM152 cells after 72 h of Cpd1 treatment (red, upregulated; green downregulated genes). Significance is indicated by FWER *p*-val < 0.05. (**b**) Overview of KDM5B-dependent regulatory pathways. Cytoscape enrichment analysis of significantly regulated gene signatures detected in RNAseq in WM3734 and patient-derived, short-term cultured CSM152 cells treated with Cpd1 over 72 h. Red node, enrichment in treatment group; blue node, decrease. The size of the nodes represents the number of genes included. (**c**) Enrichment analysis of cell cycle- and mitosis-controlling transcripts detected in WM3734$^{Tet3G\text{-}KDM5B}$ cells after 24, 48 and 72 h of Dox treatment. GSEA visualizations for each time point were combined in Cytoscape. Blue nodes represent enrichment in the control group; grey nodes were not yet present at the respective time point, the size of the nodes reflects the number of genes included. (**d**) Confirmation of KDM5B knock-down by immunoblotting. Shown is one representative (n=2). (**e**) Heatmap of Tsoi differentiation trajectory[2] from WM3734 and CSM152 cells after 12 h, 24 h and 48 h Cpd1 treatment ranked by expression at 48 h (red, upregulated; green downregulated genes). Source data are provided as a Source Data file.

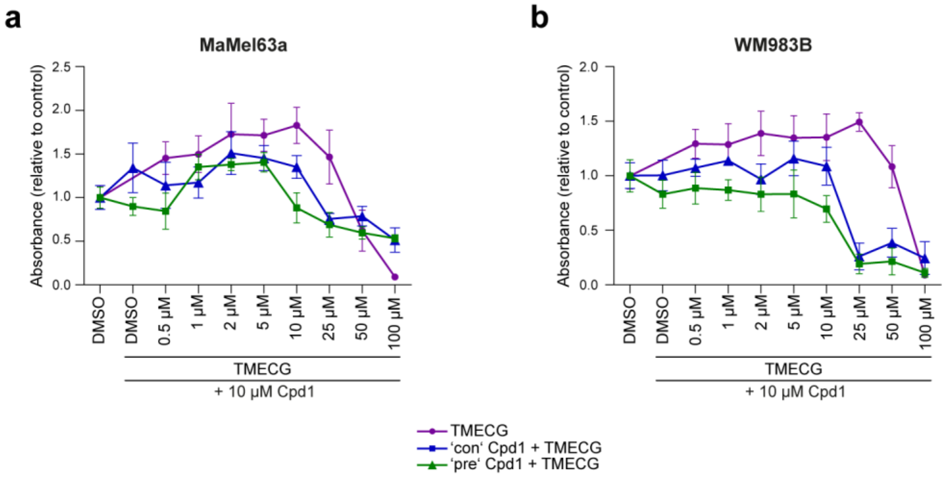

Supplementary Figure 9. Decreased cell viability upon combined Cpd1 plus TMECG treatment, Related to Figure 6. (**a and b**) MTT cell viability assays of MaMel63a (a) and WM983B (b) cells. TMECG was either concurrently given together with Cpd1 ('con') or added 3 days after Cpd1 pre-treatment ('pre'). Readout was performed after 72 h of TMECG treatment. Mean ±SD (n=3 for MaMel63a and n=2 for WM983B). Source data are provided as a Source Data file.

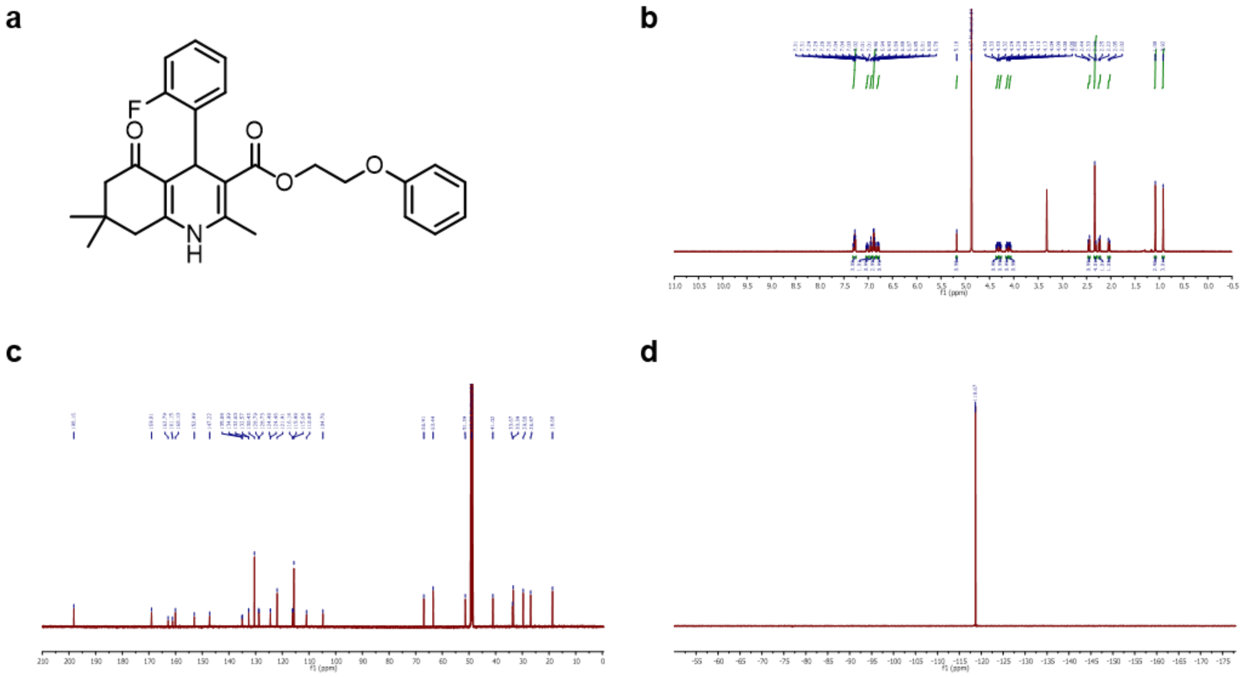

Supplementary Figure 10. Figure 9. Chemical analysis of Cpd1. Related to Material and Methods part. (**a**) Structural formula of Cpd1 (2-phenoxyethyl 4-(2-fluorophenyl)-2,7,7-trimethyl-5-oxo-1,4,5,6,7,8-hexahydroquinoline-3-carboxylate, PubChem name BAS00915510). (**b – d**) NMR analysis of Cpd1. Data are reported in the following order: chemical shift (δ) in ppm; multiplicities are indicated s (singlet), d (doublet), t (triplet), q (quartet), m (multiplet); coupling constants (J) are given in Hertz (Hz). High resolution mass spectra were recorded on a LTQ Orbitrap mass spectrometer coupled to an Accela HPLC System (HPLC column: Hypersyl GOLD, 50 mm × 1 mm, 1.9 µm). (**b**) ^{1}H-NMR of Cpd1 (600 MHz, MeOD) δ 7.29 (ddd, 1H), 7.02 (td, *J* = 7.3, 1.6 Hz, 1H), 6.94 (t, *J* = 7.3 Hz, 1H), 6.87 (dd, *J* = 16.9, 7.7 Hz, 1H), 6.79 (t, 1H), 5.18 (s, 1H), 4.34 (ddd, *J* = 12.0, 6.1, 3.3 Hz, 1H), 4.28 (ddd, *J* = 12.1, 6.2, 3.2 Hz, 1H), 4.14 (ddd, *J* = 9.5, 6.1, 3.2 Hz, 1H), 4.08 (ddd, 1H), 2.45 (d, *J* = 17.0 Hz, 1H), 2.32 (d, *J* = 15.0 Hz, 1H), 2.24 (d, *J* = 16.4 Hz, 1H), 2.04 (d, *J* = 16.4 Hz, 1H), 1.08 (s, 1H), 0.92 ppm (s, 1H). (**c**) ^{13}C-NMR (151 MHz, MeOD) δ 198.16, 169.01, 162.79, 161.15, 160.10, 152.99, 147.22, 135.03 (d, J_{CF} = 13.7 Hz), 132.59 (d, J_{CF} = 4.8 Hz), 130.43, 128.76 (d, J_{CF} = 8.4 Hz), 124.47 (d, J_{CF} = 3.3 Hz), 121.91, 116.14, 115.98, 115.64, 110.89, 104.76, 66.91, 63.44, 51.39, 41.02, 33.67, 33.39, 29.68, 26.87, 18.68 ppm. (**d**) ^{19}F-NMR (565 MHz, MeOD) δ -118.67 ppm. HR-MS: calc. for $[M+H]^+$ $C_{27}H_{29}O_4NF$ = 450.20751 found 450.20785; $C_{27}H_{28}O_4NFNa$ = 472.18946 found 472.18962.

Supplementary Table 1. Screening assay results for the selected hit (Cpd1) and control (Neg4) compound. Related to Supplementary Figure 3.

Cmpd Identifier	Structure	Mother plate (WellIndex)	PS % of K/EGFP^{high} cells mean	HC run I % of K/EGFP^{high} cells mean ±SD (n=3)	HC run II % of K/EGFP^{high} mean ±SD (n=2)	HC run III % of K/EGFP^{high} cells mean ±SD (n=3)	Mean
27849_ChemDiv _2191-2790 **(Cpd1)**	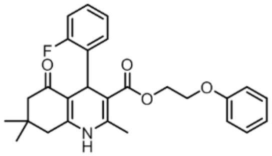	CBN_000012_D01 (Well I07)	**2.00**	0.95 ± 0.37	1.18 ± 0.24	2.27 ± 0.15	**1.47**
26798_ChemDiv _2732-4408 **(Neg4)**	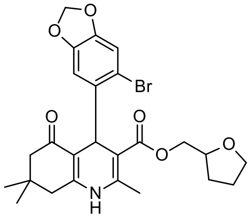	CBN_000009_D01 (Well N7)	**3.85**	no hit (n.a.)	no hit (n.a.)	no hit (n.a.)	

PS = Primary Screen
HC = Hit Confirmation

Supplementary Table 2. Normalized enrichment scores (NES) and false discovery rate (FDR) for the heatmaps of the 'Tsoi et. al differentiation signature' [2] shown in Figure 4C and Supplementary Figure 8D. Related to Figure 4 and Supplementary Figure 8.

NES	10 dox vs 0 dox (24 h)			WM3734 Cpd1 vs DMSO			CSM152 Cpd1 vs DMSO		
**Signature**	**24 h**	**48 h**	**72 h**	**12 h**	**24 h**	**48 h**	**12 h**	**24 h**	**48 h**
Undifferentiated	2.13	-1.52	-1.50	-1.38	-1.35	-1.93	-1.58	-1.14	-1.76
Undifferentiated-neural crest-like	1.27	-2.83	-2.69	-1.93	-1.86	-2.08	-2.15	-1.51	-1.74
Neural crest-like	-1.73	-2.67	-2.60	-2.26	-1.93	-1.79	-1.17	-1.64	-1.27
Neural crest-like-transitory	-1.59	1.24	1.30	-1.18	1.30	-1.42	1.15	1.31	1.17
Transitory	0.87	2.17	2.16	-1.04	1.02	-0.89	1.13	1.89	1.66
Transitory-melanocytic	-1.42	2.66	2.90	1.47	1.66	1.61	1.52	2.07	1.67
Melanocytic	0.80	1.96	2.30	-1.53	1.02	1.07	1.31	1.55	1.79

FDR	10 dox vs 0 dox(24 h)			WM3734 Cpd1 vs DMSO			CSM152 Cpd1 vs DMSO		
**Signature**	**24 h**	**48 h**	**72 h**	**12 h**	**24 h**	**48 h**	**12 h**	**24 h**	**48 h**
Undifferentiated	0.00	0.03	0.03	0.11	0.09	0.00	0.03	0.28	0.03
Undifferentiated-neural crest-like	0.25	0.00	0.00	0.01	0.00	0.00	0.00	0.04	0.02
Neural crest-like	0.02	0.00	0.00	0.00	0.00	0.01	0.24	0.03	0.14
Neural crest-like-transitory	0.04	0.16	0.12	0.25	0.27	0.07	0.32	0.11	0.24
Transitory	0.93	0.00	0.00	0.39	0.55	0.64	0.26	0.00	0.01
Transitory-melanocytic	0.06	0.00	0.00	0.05	0.06	0.02	0.10	0.00	0.01
Melanocytic	0.79	0.00	0.00	0.06	0.42	0.35	0.22	0.04	0.00

Supplementary Table 3. Primer Sequences. Related to Figure 1, 3, 6, and Supplementary Figure 3.

Name	Species	Sequence (5'-3')	Source
AURKB fwd	human	CATCACACAACGAGACCTATCGCC	
AURKB rev	human	GGGTTATGCCTGAGCAGTTTGGAG	
KDM5B all isoforms fwd	human	AACAACATGCCAGTGATGGA	3
KDM5B all isoforms rev	human	TACCAGGTTTTTGGCTCACC	3
KDM5B transcript variant 1 fwd	human	AACCTCCGCCTCCTAGATTC	
KDM5B transcript variant 1 rev	human	CGTTGTCTCCTCGGGTTCTA	
KIF4A fwd	human	GAAGAAAACCAAGGCTGAAGGGG	
KIF4A rev	human	TGGAATCTCTGTAGGGCACAAAGC	
MITF fwd	human	CCGTCTCTCACTGGATTGGT	4
MITF rev	human	TACTTGGTGGGGTTTTCGAG	4
SHCBP1 fwd	human	TGTTTGACCAGACAGCCCTTGC	
SHCBP1 rev	human	TCATCCTCCTCTTCTTCATCCCAAC	
UBE2C fwd	human	GCATCAGAACCAGCTCAACA	
UBE2C rev	human	GGTTCTGGCATTTGGAGAAA	
18S fwd	human	CGCCGCTAGAGGTGAAATTC	
18S rev	human	TCTTGGCAAATGCTTTCGC	
Myco-SE	human	GGGAGCAAACAGGATTAGATACCC	5
Myco-AS	human	TGCACCATGTGTCACTCTGTTAACCTC	5
DCT fwd	human	AACTGCGAGCGGAAGAAACC, ID 193788638c2	6
DCT rev	human	CGTAGTCGGGGTGTACTCTCT, ID 193788638c2	6
MITF fwd	human	CCGTCTCTCACTGGATTGGT, ID 8917552a1	4

MITF rev	human	TACTTGGTGGGGTTTTCGAG, ID 8917552a1	4
MLANA fwd	human	GCTCATCGGCTGTTGGTATT, ID 110625784c2	7
MLANA rev	human	TTCTTGTGGGCATCTTCTTG, ID 110625784c2	7
TYR fwd	human	GCAAAGCATACCATCAGCTCA	6
TYR rev	human	GCAGTGCATCCATTGACACAT	6

References

1. Scibetta AG, *et al.* Functional analysis of the transcription repressor PLU-1/JARID1B. *Mol Cell Biol* **27**, 7220-7235 (2007).

2. Tsoi J, *et al.* Multi-stage Differentiation Defines Melanoma Subtypes with Differential Vulnerability to Drug-Induced Iron-Dependent Oxidative Stress. *Cancer Cell* **33**, 890-904.e895 (2018).

3. Roesch A, *et al.* A temporarily distinct subpopulation of slow-cycling melanoma cells is required for continuous tumor growth. *Cell* **141**, 583-594 (2010).

4. Smith MP, *et al.* A PAX3/BRN2 rheostat controls the dynamics of BRAF mediated MITF regulation in MITF(high) /AXL(low) melanoma. *Pigment Cell Melanoma Res* **32**, 280-291 (2019).

5. Ossewaarde JM, de Vries A, Bestebroer T, Angulo AF. Application of a Mycoplasma group-specific PCR for monitoring decontamination of Mycoplasma-infected Chlamydia sp. strains. *Appl Environ Microbiol* **62**, 328-331 (1996).

6. Spandidos A, Wang X, Wang H, Seed B. PrimerBank: a resource of human and mouse PCR primer pairs for gene expression detection and quantification. *Nucleic Acids Res* **38**, D792-799 (2010).

7. Riesenberg S, *et al.* MITF and c-Jun antagonism interconnects melanoma dedifferentiation with pro-inflammatory cytokine responsiveness and myeloid cell recruitment. *Nat Commun* **6**, 8755 (2015).
