# Supplementary material for: Persister state-directed transitioning and vulnerability in melanoma
Source: Nat Commun. 2022 Jun 1;13:3055. doi: 10.1038/s41467-022-30641-9 (PMC9160289; doi:10.1038/s41467-022-30641-9)
Supplement: Supplementary file 2 — Description of Additional Supplementary Files [file 41467_2022_30641_MOESM2_ESM.pdf]

Description of Additional Supplementary Files

File Name: Supplementary Data 1
Description: Significantly regulated genes detected by mass spectrometry of WM3734 cells treated with 10 µM Cpd1 for 72 h. Related to Figure 3.

File Name: Supplementary Data 2
Description: Significantly regulated genes detected by RNAseq of cells treated with Cpd1 (WM3734 and CSM152 cells, 10 µM Cpd 1 for 72 h,) or doxycycline (WM3734Tet3G-KDM5B cells, 10 ng/ml for 24 h, 48 h or 72 h). Related to Figure 3, 4 and Supplementary Figure 8.

File Name: Supplementary Data 3
Description: Significantly regulated genes detected by RNAseq of cells treated with Cpd1 (WM3734 and CSM152 cells, 10 µM for 12 h, 24 h and 48 h). Related to Supplementary Figure 8.

File Name: Supplementary Movie 1
Description: Live cell imaging of WM3734 cells treated with DMSO as control over 5 days. Related to Figure 3.

File Name: Supplementary Movie 2
Description: Live cell imaging of WM3734 cells treated with 10 µM Neg4 as control over 5 days. Related to Figure 3.

File Name: Supplementary Movie 3
Description: Live cell imaging of WM3734 cells treated with 10 µM Cpd1 over 5 days. Related to Figure 3.
