# Supplementary material for: Persister state-directed transitioning and vulnerability in melanoma
Source: Nat Commun. 2022 Jun 1;13:3055. doi: 10.1038/s41467-022-30641-9 (PMC9160289; doi:10.1038/s41467-022-30641-9)
Supplement: Supplementary file 9 — Reporting Summary [file 41467_2022_30641_MOESM9_ESM.pdf]

nature research

Corresponding author(s): Alexander Roesch
Last updated by author(s): Mar 21, 2022

Reporting Summary

Nature Research wishes to improve the reproducibility of the work that we publish. This form provides structure for consistency and transparency in reporting. For further information on Nature Research policies, see our Editorial Policies and the Editorial Policy Checklist.

Statistics

For all statistical analyses, confirm that the following items are present in the figure legend, table legend, main text, or Methods section.

n/a	Confirmed	
☐	☒	The exact sample size (*n*) for each experimental group/condition, given as a discrete number and unit of measurement
☐	☒	A statement on whether measurements were taken from distinct samples or whether the same sample was measured repeatedly
☐	☒	The statistical test(s) used AND whether they are one- or two-sided<br>*Only common tests should be described solely by name; describe more complex techniques in the Methods section.*
☒	☐	A description of all covariates tested
☐	☒	A description of any assumptions or corrections, such as tests of normality and adjustment for multiple comparisons
☐	☒	A full description of the statistical parameters including central tendency (e.g. means) or other basic estimates (e.g. regression coefficient) AND variation (e.g. standard deviation) or associated estimates of uncertainty (e.g. confidence intervals)
☐	☒	For null hypothesis testing, the test statistic (e.g. *F*, *t*, *r*) with confidence intervals, effect sizes, degrees of freedom and *P* value noted<br>*Give P values as exact values whenever suitable.*
☒	☐	For Bayesian analysis, information on the choice of priors and Markov chain Monte Carlo settings
☒	☐	For hierarchical and complex designs, identification of the appropriate level for tests and full reporting of outcomes
☒	☐	Estimates of effect sizes (e.g. Cohen's *d*, Pearson's *r*), indicating how they were calculated

Our web collection on statistics for biologists contains articles on many of the points above.

Software and code

Policy information about availability of computer code

Data collection	TCGA data set were downloaded from UCSC Xena and Single Cell Portal (https://singlecell.broadinstitute.org). ChIP-seq data were collected from ChIP-Atlas.
Data analysis	The software used to analyze the data include: Aperio ImageScope software v12.1.0.5029, BioVinci 3.0.0, CellProfiler 3.1.8, Columbus 2.4.0, Cytoscape, FlowJo V7.6.5., Gene set enrichment analysis (GSEA- http://software.broadinstitute.org/gsea/index.jsp), GraphPad Prism v6-8, IGV browser version 2.4.9, Illumina GenomeStudio Gene Expression Module version 2009.2, ImageJ 1.48x, IMARIS 8.4, Kaluza 1.2, MaxQuant v. 1.5.3.30, Partek Expectation-Maximization algorithm, Partek Flow v6, Partek Genomic Suite v6.6, Perseus v1.5.5.1, Pre-ranked tool version 4-6.012, R v.3.1 version, StepOnePlus v2.1, UCSC Xena, VisiView® Software v5.0, Xcalibur software version 2.2. SP1.48, X-tile 3.6.1, Zen version 2.6

For manuscripts utilizing custom algorithms or software that are central to the research but not yet described in published literature, software must be made available to editors and reviewers. We strongly encourage code deposition in a community repository (e.g. GitHub). See the Nature Research guidelines for submitting code & software for further information.

Data

Policy information about availability of data

All manuscripts must include a data availability statement. This statement should provide the following information, where applicable:

- Accession codes, unique identifiers, or web links for publicly available datasets
- A list of figures that have associated raw data
- A description of any restrictions on data availability

The mass spectrometry proteomics data generated in this study have been deposited in the ProteomeXchange Consortium via the PRIDE partner repository (https://www.ebi.ac.uk/pride/archive/) under accession code PXD008757. The RNAseq data discussed in this study have been deposited in NCBI's Gene Expression

Omnibus 81 database under accession codes GSE118529 (https://www.ncbi.nlm.nih.gov/geo/query/acc.cgi?acc=GSE118529), GSE138068 (https://www.ncbi.nlm.nih.gov/geo/query/acc.cgi?acc=GSE138068) and GSE168192 (https://www.ncbi.nlm.nih.gov/geo/query/acc.cgi?acc=GSE168192). The microarray data generated in this study have been deposited in NCBI's Gene Expression Omnibus 81 database under accession code GSE137393 (https://www.ncbi.nlm.nih.gov/geo/query/acc.cgi?acc=GSE137393).
Data of The Cancer Genome Atlas TCGA data set (http://cancergenome.nih.gov/) were used for this study.
The following data sets were used for epigenetic analysis: KDM5B-ChIPseq data from breast cancer cell lines SUM185, SUM159, MCF7, HCC2157, T47D, and MDA231 (GSE46073), H3K4me3-ChIPseq data from melanoma cell lines MM27, MM13, MM16 (GSE71854) and A375 (GSE99835), and KDM5B- and H3K4me3-ChIPseq data from ChIP-Atlas (https://chip-atlas.org/).

Field-specific reporting

Please select the one below that is the best fit for your research. If you are not sure, read the appropriate sections before making your selection.

☒ Life sciences ☐ Behavioural & social sciences ☐ Ecological, evolutionary & environmental sciences

For a reference copy of the document with all sections, see nature.com/documents/nr-reporting-summary-flat.pdf

Life sciences study design

All studies must disclose on these points even when the disclosure is negative.

Sample size	(i) Animal experiments: The numbers were calculated based on Kaplan-Meier-Survival analysis based on tumor size. The calculation was performed with the publicaly available software-Tool "StatsToDo". (ii) In vitro cell culture experiments: No sample size calculations were performed. Sample sizes are indicated in the figure legends. Sample size was determined based on preliminary experiments that defined the adequate number of samples to consistently identify differences between groups.
Data exclusions	Single data points were only excluded in case of documented technical reasons such as contamination of a single well in a 6-fold MTT replicate or if identified as outlayer in GraphPad Prism.
Replication	For all experiments, replication experiments were succesful and showed comparable results. Regarding replication of key tools for KDM5B upscaling, the in vitro experiments with the Tet-On 3G-KDM5B cell lines were more than 3 times sucessfully replicated including use of different clones. In vivo, we used three different mouse models to reproduce the effect of enforced KDM5B upscaling (WM3734 xenograft + Tet-On 3G, WM3734 + Cpd1 and CM syngeneic + Cpd1, one experiment each). The results of all mouse models were comparable. Mouse group sizes are indicated in Figure legends.
Randomization	Mice were randomized according to cage number and tumor burden. If drugs were provided in drinking water, mice were randomized before drug treatment in different cages. However, in one cage only one treatment was applied. For in vitro experiments, no relevant differing covariates for cell lines could be identified.
Blinding	For in vivo work, investigators were not totally blinded to group allocation, because of the rules of the animal facility that each cage has to be labeled with the treatment applied. However, for measurment one person picked the cage and another person measured tumor size without knowing which exact cage/treamtent. For bioinformatic analysis, molecular and biochemical data analyses, blinding was not performed. During scoring or counting of cell-based assays, investigators were blinded.

Reporting for specific materials, systems and methods

We require information from authors about some types of materials, experimental systems and methods used in many studies. Here, indicate whether each material, system or method listed is relevant to your study. If you are not sure if a list item applies to your research, read the appropriate section before selecting a response.

Materials & experimental systems

n/a	Involved in the study	
☐	☒	Antibodies
☐	☒	Eukaryotic cell lines
☒	☐	Palaeontology and archaeology
☐	☒	Animals and other organisms
☒	☐	Human research participants
☒	☐	Clinical data
☒	☐	Dual use research of concern

Methods

n/a	Involved in the study	
☒	☐	ChIP-seq
☐	☒	Flow cytometry
☒	☐	MRI-based neuroimaging

Antibodies

Antibodies used	The following antibodies were used in this study:<br>Alexa Fluor 488 goat anti-mouse, ThermoFisher Scientific, Cat# A11011;<br>Alexa Fluor® 647-AffiniPure F(ab')2 Fragment Goat Anti-Rabbit IgG (H+L), Dianova, Cat# 111-606-045;

Anti-FKBP12, Abcam,Cat# ab24373;
Anti-Histone H3 antibody, Abcam, Cat# ab1791;
Anti-Histone H3 (tri methyl K4) antibody, Abcam, Cat# ab8580;
Anti-MITF antibody, Abcam, Cat# ab12039;
Anti-MITF antibody, Sigma, Cat# M6065;
Anti-α-Tubulin antibody, Abcam, Cat# ab18251;
α/ß-Tubulin antibody, Cell Signaling, Cat# 2148;
AXL antibody, Cell Signaling, Cat#8661;
GAPDH antibody, Santa Cruz, Cat# SC-25778;
GOAT ANTI-RABBIT IGG (H+L) CROSS-ADSORBED SECONDARY ANTIBODY, ALEXA FLUOR 568, ThermoFisher Scientific, Cat# A11011;
Lysine (K)-specific Demethylase 5B/KDM5B/JARID1B antibody, Novus Biologicals, Cat# NB100-97821;
N-Cadherin (D4R1H) XP Rabbit, Cell Signaling, Cat# 13116;
Peroxidase AffiniPure Goat Anti-Rabbit IgG, Fc Fragment Specific, Jackson ImmunoResearch Laboratories, Cat# 115-035-046;
Peroxidase AffiniPure Goat Anti-Mouse IgG (H+L), Jackson ImmunoResearch Laboratories, Cat# 115-035-003;
Purified Mouse Anti- AIM-1 Clone 6/AIM-1 (RUO), BD Biosciences, Cat# 611082;
Rabbit polyclonal IgG, Dianova, Cat# DLN-13122;
TCF8/ZEB1 (D80D3) Rabbit mAb, Cell Signaling, Cat# 3396;
Smad1 Antibody, Cell Signaling, Cat# 9743;
ZO-1 (D7D12) Rabbit mAb, Cell Signaling, Cat# 8193

Validation

The antibodies used are well described and validated and were pruchased from well-reputable companies.
Alexa Fluor 488 goat anti-mouse, ThermoFisher Scientific, Cat# A11011; https://www.thermofisher.com/antibody/product/Goat-anti-Mouse-IgG-H-L-Cross-Adsorbed-Secondary-Antibody-Polyclonal/A-11001, suitable for FACS, IF
Alexa Fluor® 647-AffiniPure F(ab')2 Fragment Goat Anti-Rabbit IgG (H+L), Dianova, Cat# 111-606-045; https://www.jacksonimmuno.com/catalog/products/111-606-045, suitable for FACS, IF
Anti-FKBP12, Abcam,Cat# ab24373; https://www.abcam.com/fkbp12-antibody-ab24373.html; suitable for WB
Anti-Histone H3 antibody, Abcam, Cat# ab1791; https://www.abcam.com/histone-h3-antibody-nuclear-marker-and-chip-grade-ab1791.html; suitable for WB
Anti-Histone H3 (tri methyl K4) antibody, Abcam, Cat# ab8580; https://www.abcam.com/histone-h3-tri-methyl-k4-antibody-chip-grade-ab8580.html, suitable for WB
Anti-MITF antibody, Abcam, Cat# ab12039; https://www.abcam.com/mitf-antibody-c5-ab12039.html, suitable for IF, IHC, WB
Anti-MITF antibody, Sigma, Cat# M6065; https://www.sigmaaldrich.com/DE/de/product/sigma/m6065, suitable for IF, IHC, WB
Anti-α-Tubulin antibody, Abcam, Cat# ab18251; https://www.abcam.com/alpha-Tubulin-antibody-Microtubule-Marker-ab18251.html?gclsrc=aw.ds|aw.ds&gclid=EAIaIQobChMIz4biicrF9gIVTQKLCh3KOgYMEAAYASAAEgLvmvD_BwE, suitable for IF
α/ß-Tubulin antibody, Cell Signaling, Cat# 2148; https://www.cellsignal.com/products/primary-antibodies/a-b-tubulin-antibody/2148, suitable for WB
AXL antibody, Cell Signaling, Cat#8661; https://www.cellsignal.com/products/primary-antibodies/axl-c89e7-rabbit-mab/8661, suitable for WB
GAPDH antibody, Santa Cruz, Cat# SC-25778; https://www.scbt.com/de/p/gapdh-antibody-fl-335, suitable for WB
GOAT ANTI-RABBIT IGG (H+L) CROSS-ADSORBED SECONDARY ANTIBODY, ALEXA FLUOR 568, ThermoFisher Scientific, Cat# A11011; https://www.thermofisher.com/antibody/product/Goat-anti-Rabbit-IgG-H-L-Cross-Adsorbed-Secondary-Antibody-Polyclonal/A-11011, suitable for IF
Lysine (K)-specific Demethylase 5B/KDM5B/JARID1B antibody, Novus Biologicals, Cat# NB100-97821; https://www.novusbio.com/products/lysine-k-specific-demethylase-5b-kdm5b-jarid1b-antibody_nb100-97821, suitable for FACS, IF, IHC, WB
N-Cadherin (D4R1H) XP Rabbit, Cell Signaling, Cat# 13116; https://www.cellsignal.com/products/primary-antibodies/n-cadherin-d4r1h-xp-rabbit-mab/13116, suitable for WB
Peroxidase AffiniPure Goat Anti-Rabbit IgG, Fc Fragment Specific, Jackson ImmunoResearch Laboratories, Cat# 115-035-046; https://www.jacksonimmuno.com/catalog/products/111-035-046, suitable for WB
Peroxidase AffiniPure Goat Anti-Mouse IgG (H+L), Jackson ImmunoResearch Laboratories, Cat# 115-035-003; https://www.jacksonimmuno.com/catalog/products/115-035-003, suitable for WB
Purified Mouse Anti- AIM-1 Clone 6/AIM-1 (RUO), BD Biosciences, Cat# 611082; https://www.citeab.com/antibodies/2412175-611082-bd-transduction-laboratories-purified-mouse, suitable for IF
Rabbit polyclonal IgG, Dianova, Cat# DLN-13122; suitable for IF, IHC
TCF8/ZEB1 (D80D3) Rabbit mAb, Cell Signaling, Cat# 3396; https://www.cellsignal.com/products/primary-antibodies/zeb1-d80d3-rabbit-mab/3396, suitable for WB
Smad1 Antibody, Cell Signaling, Cat# 9743; https://www.cellsignal.com/products/primary-antibodies/smad1-antibody/9743, suitable for WB
ZO-1 (D7D12) Rabbit mAb, Cell Signaling, Cat# 8193, https://www.cellsignal.com/products/primary-antibodies/zo-1-d7d12-rabbit-mab/8193, suitable for WB

Eukaryotic cell lines

Policy information about cell lines

Cell line source(s)

The cell lines 451Lu, 451Lu BR, WM164, WM3734, WM88, WM9, WM983B and WM983B BR were obtained from the Wistar Institute. The cell lines MelJuSo, MeWo, SKMel5 and SKMel28 were obtained from the ATCC. The primary patient-derived melanoma cell lines CSM027, CSM152, ES014028 fibroblasts and the murine melanoma cell lines CM and MaMel63a were established at the Department of Dermatology of the University Hospital Essen.

Authentication

Cell line identity was confirmed by PCR-based DNA fingerprinting at the Department of Pathology of the University Hospital Essen.

Mycoplasma contamination	All cell lines were tested negative for mycoplasma contamination.
Commonly misidentified lines (See ICLAC register)	None of the commonly misidentified lines were used.

Animals and other organisms

Policy information about studies involving animals; ARRIVE guidelines recommended for reporting animal research

Laboratory animals	6-8 week old NMRI-(nu/nu)-nude mice (both sex) and 6-8 week old female C57BL/6 mice were used for experiments. All mice were housed in rooms maintained at a constant temperature of 22°C and 45-65% humidity with a 12 hour light cycle. Animals were allowed food and water ad libitum.
Wild animals	The study did not involve wild animals.
Field-collected samples	The study did not involve samples collected at the field.
Ethics oversight	All animal experiments were performed in accordance with institutional and national guidelines and regulations. The protocols have been approved by the Wistar IACUC protocol 111954 or the local German authority Landesamt für Natur, Umwelt und Verbraucherschutz Nordrhein-Westfalen – LANUV NRW in compliance with the German animal protection law (Reference number AZ 84-02.04.2014.A08 AZ 81-02.04.2018.A202). Studies on human tissue samples and establishment of human melanoma cell lines were approved by the Internal Review Boards of the University of Pennsylvania School of Medicine and The Wistar Institute or the ethics committees of the Medical Faculties of the University of Wuerzburg and the University of Duisburg-Essen (reference numbers: 123/08_ff, 11-4715, 17-7391-BO).

Note that full information on the approval of the study protocol must also be provided in the manuscript.

Flow Cytometry

Plots

Confirm that:

☒ The axis labels state the marker and fluorochrome used (e.g. CD4-FITC).

☒ The axis scales are clearly visible. Include numbers along axes only for bottom left plot of group (a 'group' is an analysis of identical markers).

☒ All plots are contour plots with outliers or pseudocolor plots.

☒ A numerical value for number of cells or percentage (with statistics) is provided.

Methodology

Sample preparation	Cells were treated in cell culture and collcected by trypsinisation. Either cells were fixed for intracellular stainings or stained without fixation.
Instrument	Gallios flow cytometer (Beckman Coulter, Brea, CA, USA)
Software	Data were collected and analyzed with Kaluza 1.2 (Beckman Coulter, Brea, CA, USA) or FlowJo V7.6.5 (Tree Star, Ashland, Oregon, USA) software.
Cell population abundance	NA, since we only performed analytical flow cytometer and did not perform cell sorting.
Gating strategy	In all experiments living cells were gated by FSC/SSC scatter. For intracellular KDM5B staining gates were set based on the DMSO control, which was set to 1% KDM5Bhigh cells. For quantitation of the K/EGFP reporter construct, a 5%-threshold for the K/EGFP signal intensity was applied as described previously. PI staining was measured in linear scale.

☒ Tick this box to confirm that a figure exemplifying the gating strategy is provided in the Supplementary Information.
